# Molecular Profiling of Inflammatory Processes in a Mouse Model of IC/BPS: From the Complete Transcriptome to Major Sex-Related Histological Features of the Urinary Bladder

**DOI:** 10.3390/ijms24065758

**Published:** 2023-03-17

**Authors:** Dominika Peskar, Tadeja Kuret, Katja Lakota, Andreja Erman

**Affiliations:** 1 Institute of Cell Biology, Faculty of Medicine, University of Ljubljana, 1000 Ljubljana, Slovenia; 2 Department of Rheumatology, University Medical Center Ljubljana, 1000 Ljubljana, Slovenia

**Keywords:** interstitial cystitis/bladder pain syndrome, inflammation, RNA sequencing, animal model, cyclophosphamide

## Abstract

Animal models are invaluable in the research of the pathophysiology of interstitial cystitis/bladder pain syndrome (IC/BPS), a chronic aseptic urinary bladder disease of unknown etiology that primarily affects women. Here, a mouse model of IC/BPS was induced with multiple low-dose cyclophosphamide (CYP) applications and thoroughly characterized by RNA sequencing, qPCR, Western blot, and immunolabeling to elucidate key inflammatory processes and sex-dependent differences in the bladder inflammatory response. CYP treatment resulted in the upregulation of inflammatory transcripts such as *Ccl8*, *Eda2r*, and *Vegfd*, which are predominantly involved in innate immunity pathways, recapitulating the crucial findings in the bladder transcriptome of IC/BPS patients. The JAK/STAT signaling pathway was analyzed in detail, and the JAK3/STAT3 interaction was found to be most activated in cells of the bladder urothelium and lamina propria. Sex-based data analysis revealed that cell proliferation was more pronounced in male bladders, while innate immunity and tissue remodeling processes were the most distinctive responses of female bladders to CYP treatment. These processes were also reflected in prominent histological changes in the bladder. The study provides an invaluable reference dataset for preclinical research on IC/BPS and an insight into the sex-specific mechanisms involved in the development of IC/BPS pathology, which may explain the more frequent occurrence of this disease in women.

## 1. Introduction

Interstitial cystitis/bladder pain syndrome (IC/BPS) is a chronic aseptic inflammatory disease that primarily affects the urinary bladder and is characterized by increased urination frequency and chronic pain or discomfort in the pelvic region [1]. The diagnosis of IC/BPS is usually based on the exclusion of other conditions, such as overactive bladder syndrome and chronic prostatitis, which often overlap. The cystoscopy findings in the bladders of the patients are frequently unspecific, with the exception of Hunner lesions—denudations of urinary bladder epithelium (urothelium), however, they can be found in only a fraction of patients [2]. In contrast, the majority of patients show no histopathologic changes in the bladder (non-Hunner type of IC/BPS). Setting the right diagnosis of both types of IC/BPS remains challenging, and as a consequence, the disease is gravely underdiagnosed [1,3]. In addition, current therapeutic approaches for IC/BPS mostly aim to relieve the pain through behavioral alterations and medication, but rarely provide a complete resolution of symptoms [1]. To date, there is no therapy that could cure the disease or offer a complete alleviation of the symptoms in all patients. This may be related to the lack of understanding of the pathophysiology of IC/BPS, which still remains elusive. Since the obtainment of bladder specimens is a highly invasive procedure for the patients, progress in the elucidation of mechanisms involved in the development of IC/BPS is hindered. Therefore, animal models play a crucial role in preclinical studies of the disease and enable the discovery of novel biomarkers and therapeutic targets [4,5]. The most commonly used animal model of IC/BPS is cyclophosphamide (CYP)-induced bladder inflammation in rodents, which mimics the bladder-centric features of the disease such as inflammation, pain, increased voiding frequency, and increased urothelial permeability [6,7,8]. Therefore, understanding the model itself is very important for the translation of obtained data to the human condition.

Following systemic application of CYP, a toxic metabolite of CYP, acrolein, is secreted by urine, causing injury to the bladder mucosa while stored in the bladder [9]. In general, CYP is used either to induce acute cystitis, where hemorrhages, massive destruction of mucosa, and extensive infiltration of inflammatory cells are achieved after a single application of a higher dose [8], or to induce chronic cystitis by multiple injections of lower concentrations of CYP [6,7]. The chronic model is characterized by increased voiding frequency, reduced voiding volume, minimal weight loss, edema of the lamina propria (LP), inflammatory cell infiltration, and no urothelial ulcerations [6] and is therefore a better representation of the IC/BPS-specific bladder changes.

Cytokines and chemokines may play an important role in the pathogenesis of IC/BPS as modulators of the inflammatory microenvironment of the bladder. Changes in cytokine profiles in the urine and serum of IC/BPS patients have previously been established [10,11]. The cytokines act as the main mediators of various innate immunity pathways, such as TLR, TNF, and JAK/STAT signaling pathways, which have also been demonstrated to be enriched in the bladders of IC/BPS patients, especially in the context of chemotaxis of inflammatory cells [12,13,14]. Apart from that, the role of innate immunity in IC/BPS development remains poorly understood.

The prevalence of IC/BPS has been reported to vary based on race, geographic region, and disease awareness [3,15]. However, the women-to-men ratio in incidence of the disease is considered to be approximately 5:1 [16]. Additionally, significantly greater severity of urinary symptoms, pain intensity, and pain extent have been reported in women compared to men with IC/BPS [17]. Despite the growing evidence of sex-dependent symptom occurrence and severity, animal models of IC/BPS as well as studies of human bladder samples rarely include subjects of both sexes.

**Figure 1:**
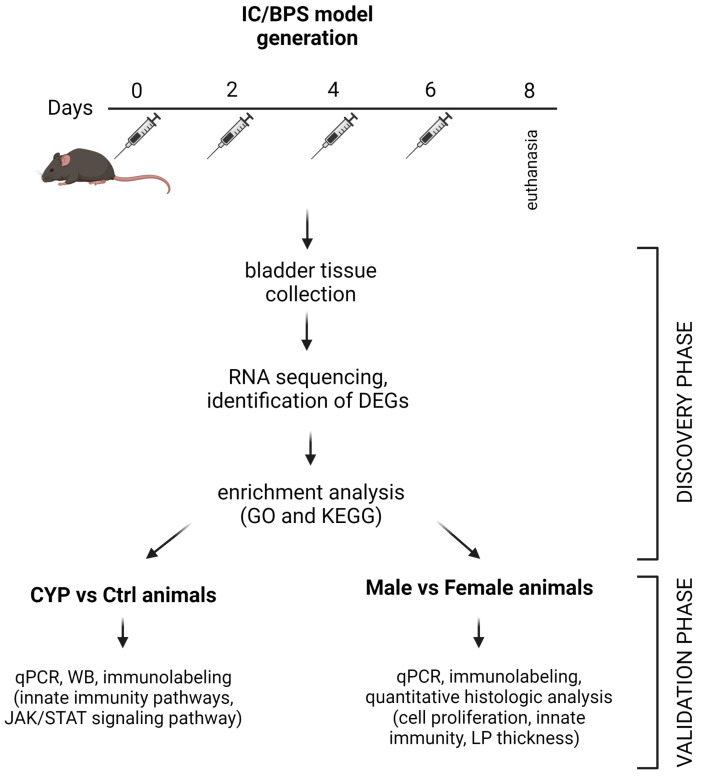
Representation of study workflow. Created with www.biorender.com (accessed on 20 January 2023).

In the present study, an IC/BPS model was generated in C57BL/6J mice that were repeatedly injected with a low CYP dose over a longer period of time, mimicking the chronicity of the disease itself (Figure 1). Here, we used animals of both sexes to examine basic differences in the bladder response to CYP administration between females and males, which could contribute to understanding the mechanisms that predispose women to the development of IC/BPS. For these purposes, we obtained complete transcriptomic data of coding RNAs from the urinary bladders using RNA sequencing (RNA seq) technology, which was subsequently validated using other methods (e.g., qPCR, Western blot, immunolabeling) with an emphasis on inflammatory processes. Overall, our study provides an in-depth analysis of the CYP-induced chronic cystitis model and uncovers the importance of sex-dependent differences in bladder response to pro-inflammatory stimuli. These differences may potentially explain the different incidence and severity of IC/BPS in female and male patients and provide new data for an advanced diagnostic and therapeutic approach in the future.

## 2. Results

### 2.1. CYP Treatment Significantly Alters the Transcriptome Profile of Urinary Bladders

**Figure 2:**
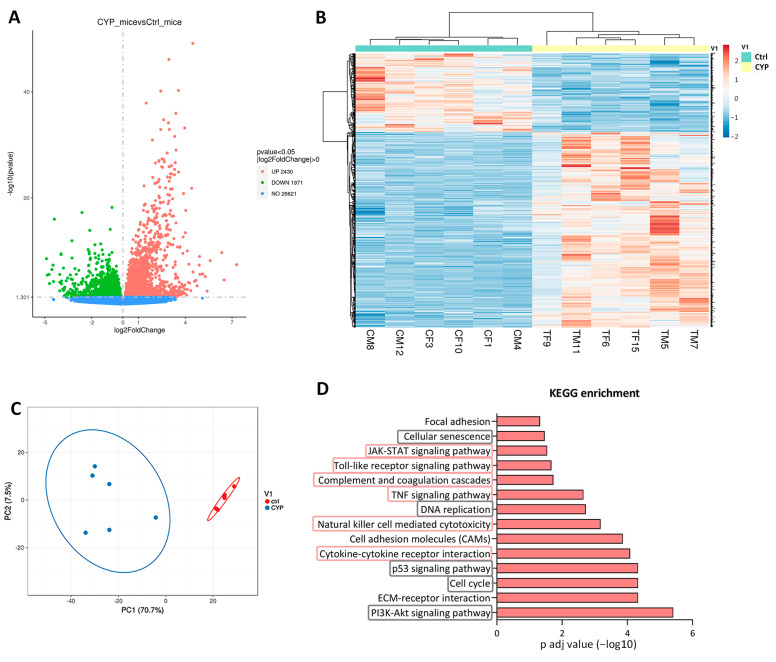
Effects of CYP treatment on the complete transcriptome of mouse bladders. (**A**) A volcano plot showing upregulated (red), downregulated (green), and unchanged (blue) genes in CYP vs. Ctrl mice. (**B**) Heatmap showing the 1000 most significantly DEGs in CYP (yellow; *n* = 6) vs. Ctrl (green; *n* = 6) animals (*p* adj < 0.05). Both rows and columns are clustered using correlation distance and average linkage. Lower expression is indicated in blue, and higher expression is indicated in red. CM: Ctrl male; CF: Ctrl female; TM: CYP-treated male; TF: CYP-treated female. (**C**) PCA of the 1000 most significantly DEGs in CYP (blue; *n* = 6) vs. Ctrl (red; *n* = 6) animals (*p* adj < 0.05), showing principal components 1 and 2 (PC1 and PC2) explaining 70.7% and 7.5% of the total variance, respectively. Unit variance scaling was applied to rows, and singular value decomposition (SVD) with imputation was used to calculate principal components. The prediction ellipses are such that with a probability of 0.95, a new observation from the same group will fall inside the ellipse. (**D**) KEGG enrichment analysis based on all DEGs in CYP (*n* = 6) vs. Ctrl (*n* = 6) animals (*p* adj < 0.05), showing relevant significantly enriched pathways, represented as −log10 of the *p* adj value. Processes involved in cell proliferation are circled in gray, while processes of immune response are circled in pink.

In order to characterize the transcriptome of the bladders after inducing inflammation by CYP administration, we first performed a comprehensive transcriptome profiling using RNA seq technology in bladder samples from 6 Ctrl animals (3 males, 3 females) and 6 CYP-treated animals (3 males, 3 females). Among the 31,022 genes aligned to the reference genome in all samples, 4401 were identified as DEG (*p* < 0.05) in CYP animals vs. Ctrl animals, of which 2430 were upregulated and 1971 were downregulated. Upregulated genes exhibited higher fold changes and *p*-adjusted values (*p* adj) than downregulated genes. (Figure 2A). Additionally, unsupervised hierarchical clustering based on the top 1000 most significantly DEGs (*p* adj < 0.05) showed a clear distinction between the CYP and Ctrl groups based on the similarities in gene expression patterns (Figure 2B). PCA of the top 1000 significantly DEGs also indicated a clear separation between the CYP and Ctrl groups, with PC1 and PC2 covering approximately 70% and 8% of the variation, respectively, suggesting that the CYP treatment is the main source of variance between samples (Figure 2C).

### 2.2. The Transcriptome of CYP-Treated Bladders Is Enriched in Processes of Immune Response and Cell Proliferation

To elucidate the functions, biological processes, and pathways involving DEGs identified in our CYP-treated mouse samples, KEGG and GO enrichment analyses were performed (complete data is available in Tables S4 and S5).

KEGG analysis comparing all DEGs (up- and downregulated) detected in CYP vs. Ctrl groups of animals identified 54 significantly enriched pathways (*p* adj < 0.05). After the exclusion of pathways connected with infectious diseases, non-bladder-related pathologies, and cancer, 14 relevant enriched pathways remained, mostly involved in cell cycle regulation or immune response (Figure 2D). The enriched pathways (listed from most to least significant) included the PI3K-Akt signaling pathway, extracellular matrix (ECM)-receptor interaction, cell cycle, p53 signaling pathway, cytokine-cytokine receptor interaction, cell adhesion molecules, natural killer cell-mediated cytotoxicity, DNA replication, TNF signaling pathway, complement and coagulation cascades, Toll-like receptor signaling pathway, JAK-STAT signaling pathway, cellular senescence, and focal adhesion (Figure 2D). In the further analysis, we focused on the validation of inflammation-related processes since chronic inflammation is a characteristic of IC/BPS and the currently available therapy mainly targets the alleviation of inflammation and pain.

### 2.3. CYP Treatment Activates the Innate Immune Response of the Bladder

**Figure 3:**
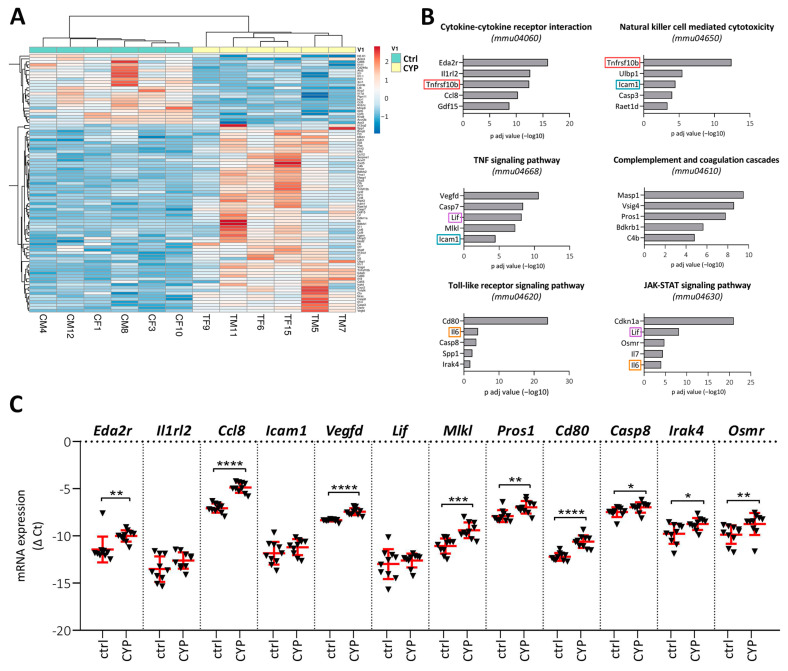
Effects of CYP treatment on the innate immune response in mouse bladders. (**A**) Heatmap showing DEGs (*n* = 89) with *p* adj < 0.05 detected in the enriched KEGG pathways of innate immunity response comparing the CYP (yellow; *n* = 6 animals) vs. Ctrl (green; *n* = 6 animals) groups. Both the rows and columns are clustered using correlation distance and average linkage. Lower expression is marked in blue; higher expression is marked in red. CM: Ctrl male; CF: Ctrl female; TM: CYP-treated male; TF: CYP-treated female. (**B**) A graphical representation of the five most significantly DEGs in each enriched pathway of innate immunity. Shown are the −log10 of the *p* adj values of the transcripts in the CYP vs. Ctrl group of animals. Circled are DEGs enriched in more than one pathway. (**C**) qPCR validation of the selected DEGs shown in (**B**), confirming their upregulation in the CYP vs. Ctrl group. Shown are the means ± SD of negative Δ Ct determined for 10 animals per group. The qPCR analysis was not reliable for some DEGs (*Tnfrsf10b*, *Gdf15*, *Ulbp1*, *Casp3*, *Raet1d*, *Casp7*, *Masp1*, *Vsig4*, *Bdkrb1*, *C4b*, *Il6*, *Spp1*, *Cdkn1a*, *Il7*), therefore the results are not presented. * *p* < 0.05; ** *p* < 0.01; *** *p* < 0.001; **** *p* < 0.0001.

The role of innate immunity is indispensable in lower urinary tract infections [18], but its importance in the pathophysiology of IC/BPS is only recently gaining more insight [14,19]. In our study, many of the identified DEGs (with *p* < 0.05) were enriched in innate immunity-related pathways (Figure 2D), such as cytokine-cytokine receptor interaction (containing 80 DEGs), natural killer cell-mediated cytotoxicity (39 DEGs), TNF signaling (39 DEGs), complement and coagulation cascade (27 DEGs), TLR signaling (29 DEGs), and JAK/STAT signaling (42 DEGs). These innate immunity-related significantly DEGs (*p* adj < 0.05; 89 altogether) were again subjected to unsupervised hierarchical clustering analysis. A clear distinction between CYP-treated and Ctrl animals, with the majority of DEGs upregulated in the CYP group, suggests upregulation of these pathways in the CYP model (Figure 3A). Figure 3B shows p adj values for the five most significantly enriched DEGs in each pathway. Some of the DEGs were enriched in more than one pathway.

Next, we aimed to validate the differential expression of the most significantly DEGs by qPCR. We confirmed CYP-induced upregulation of the majority of the analyzed DEGs, specifically *Eda2r* (*p* = 0.0014), *Ccl8* (*p* < 0.0001), *Vegfd* (*p* < 0.0001), *Mlkl* (*p* = 0.0001), *Pros1* (*p* = 0.0052), *Cd80* (*p* < 0.001), *Casp8* (*p* = 0.0185), *Irak4* (*p* = 0.0156), and *Osmr* (*p* = 0.0089). In contrast, we did not confirm the significant difference for *Il1rl2*, *Icam1*, and *Lif* (Figure 3C).

#### 2.3.1. CYP Treatment Activates the JAK3/STAT3 Signaling Pathway in the Bladder

One of the identified enriched pathways of innate immunity in our transcriptomic data included the Janus kinase/signal transducer and activator of transcription (JAK/STAT) signaling pathway (Figure 2D). JAKs are non-receptor tyrosine protein kinases that activate upon the binding of cytokines to their putative receptors. In turn, the STATs are phosphorylated, dimerized, and transported into the nucleus, where they act as regulators of transcription. Their activity is regulated by suppressors of cytokine signaling (SOCS) and protein tyrosine phosphatases (PTP), among others [20,21]. The involvement of JAK/STAT signaling in the pathophysiology of various diseases has already been extensively reported [20,21], but its role in the development of IC/BPS remains poorly understood. Therefore, we sought to explore JAK/STAT signaling in detail in the CYP-induced mouse model of IC/BPS.

**Figure 4:**
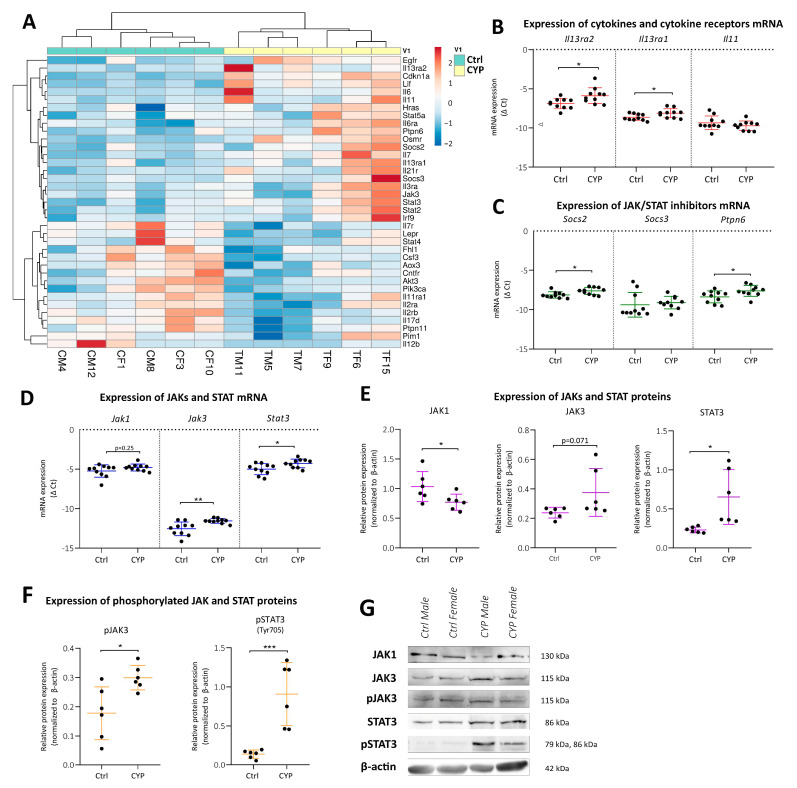
The JAK/STAT signaling pathway is activated in the bladders of CYP-treated animals. (**A**) Heatmap showing DEGs (*n* = 37) with *p* adj < 0.05 detected in the JAK/STAT signaling pathway by KEGG analysis comparing the CYP (yellow; *n* = 6 animals) vs. Ctrl (green; *n* = 6 animals) groups. Both the rows and columns are clustered using correlation distance and average linkage. Lower expression is marked in blue, and higher expression is marked in red. CM: Ctrl male; CF: Ctrl female; TM: CYP-treated male; TF: CYP-treated female. (**B**) qPCR validation of mRNA expression for cytokines and cytokine receptors (*Il13ra2*, *Il13ra1*, *Il11*), comparing CYP and Ctrl animals. Shown are the mean ± SD of negative Δ Ct determined for 10 animals per group. The qPCR analysis was not reliable for some DEGs identified as cytokines and cytokine receptors in the JAK/STAT signaling pathway (*Il6*, *Il6ra*, *Il7*, *Il21r*, *Il3ra*), therefore the results are not presented. (**C**) qPCR validation of mRNA expression for inhibitors of JAK/STAT signaling (*Socs2*, *Socs3*, *Ptpn6*), comparing CYP and Ctrl animals. Shown are the means ± SD of negative Δ Ct determined for 10 animals per group. (**D**) qPCR validation of transcript expression for the analyzed JAK and STAT molecules (*Jak1*, *Jak3*, *Stat3*), comparing CYP and Ctrl animals. Shown are the means ± SD of negative Δ Ct determined for 10 animals per group. (**E**) Expression of JAK and STAT proteins (JAK1, JAK3, and STAT3), determined by Western blot in Ctrl and CYP animals. Shown are the mean ± SD of relative protein expression normalized to β-actin for six animals per group. (**F**) The expression of phosphorylated JAK and STAT proteins (pJAK3, pSTAT3), determined by Western blot in Ctrl and CYP animals. Shown are the mean ± SD of relative protein expression normalized to β-actin for six animals per group. (**G**) Representative blots for the analysis of protein expression in six animals per group. * *p* < 0.05; *** *p* < 0.001.

The significantly DEGs (*p* adj < 0.05) involved in the JAK/STAT signaling pathway (37 altogether) were first subjected to unsupervised hierarchical clustering analysis, which showed a clear distinction between the CYP and Ctrl animals (Figure 4A). The majority of upregulated DEGs involved in the JAK/STAT pathway encode (i) cytokines or cytokine receptors (*Il13ra2*, *Lif*, *Il6*, *Il11*, *Il6ra*, *Osmr*, *Il7*, *Il13ra1*, *Il21r*, *Il3ra*), (ii) JAK/STAT molecules (*Stat5a*, *Jak3*, *Stat3*, *Stat2*), or (iii) inhibitors of JAK/STAT signaling (*Ptpn6*, *Socs2*, *Socs3*) [21]. We performed qPCR analysis to validate the DEGs identified by RNA seq encoding cytokines, cytokine receptors, and JAK/STAT inhibitors. Significantly higher mRNA expression was confirmed for *Il13ra2* (*p* = 0.0147), *Il13ra1* (*p* = 0.0175), and *Osmr* (*p* = 0.0089), but not *Lif* or *Il11*, in CYP compared to Ctrl animals (Figure 3C and Figure 4B). Among the inhibitors of JAK/STAT signaling, the significant increase in mRNA expression was confirmed for *Socs2* (*p* = 0.011) and *Ptpn6* (*p* = 0.027) in CYP compared to Ctrl animals, while no differences were detected for *Socs3* (Figure 4C).

In addition to qPCR analysis, we also examined the protein level and activation status (indicated by phosphorylation) of JAK and STAT molecules by Western blot. *Jak3* mRNA expression was significantly higher in CYP animals compared to Ctrl animals (*p* = 0.008), whereas JAK3 protein expression showed a similar trend but did not reach significance (Figure 4D,E,G). The expression of phosphorylated JAK3 was significantly increased in CYP vs. Ctrl animals (*p* = 0.013; Figure 4F,G), indicating its activation during bladder inflammation. Although our RNA seq database showed no difference in the expression of *Jak1* between CYP and Ctrl animals, we still included it in the validation analysis since it is widely expressed in various tissues and can phosphorylate all STATs [21]. Interestingly, there were no differences in the mRNA expression of *Jak1* between the CYP and Ctrl groups (Figure 4D), while the expression of JAK1 protein was significantly reduced in CYP vs. Ctrl animals (*p* = 0.048; Figure 4E,G). pJAK1 was undetectable by Western blot, which may indicate a fast dephosphorylation of the protein by the corresponding PTPs [21,22]. Despite higher basal values of *Jak1* vs. *Jak3* mRNA in the bladders of Ctrl animals, it appears that the JAK/STAT pathway is not activated via JAK1 in CYP-induced cystitis.

RNA seq showed upregulation of *Stat2*, *Stat3*, and *Stat5a* transcripts. Our preliminary Western blot data showed a bladder-specific signal only for STAT3, which we analyzed in detail as a key downstream molecule of JAK3 activation. mRNA and protein expression of *Stat3* were significantly higher in CYP vs. Ctrl animals (*p* = 0.024 and *p* = 0.015, respectively; Figure 4D,E,G). The activated form of the protein—pSTAT3 phosphorylated at Tyr705—was also significantly increased in CYP vs. Ctrl animals (*p* = 0.0009; Figure 4F,G). We suggest that JAK3-induced phosphorylation of STAT3 is the key interaction of the JAK/STAT signaling pathway in the mouse bladders after CYP treatment. However, translocation of pSTAT3 to the nucleus is crucial for the regulation of transcription.

#### 2.3.2. pSTAT3 Acts as a Key Transcriptional Factor in the CYP-Treated Bladder Urothelial Cells, Fibroblasts, and Macrophages

For signal transduction and downstream biological effects, phosphorylated STATs must translocate to the nucleus and bind to DNA as transcription factors [21]. Since we identified STAT3 as a key transcriptional factor in the JAK/STAT signaling pathway, we wanted to investigate whether nuclear translocation of pSTAT3 occurs due to CYP treatment and which cells of the urinary bladder have the highest nuclear expression of pSTAT3.

**Figure 5:**
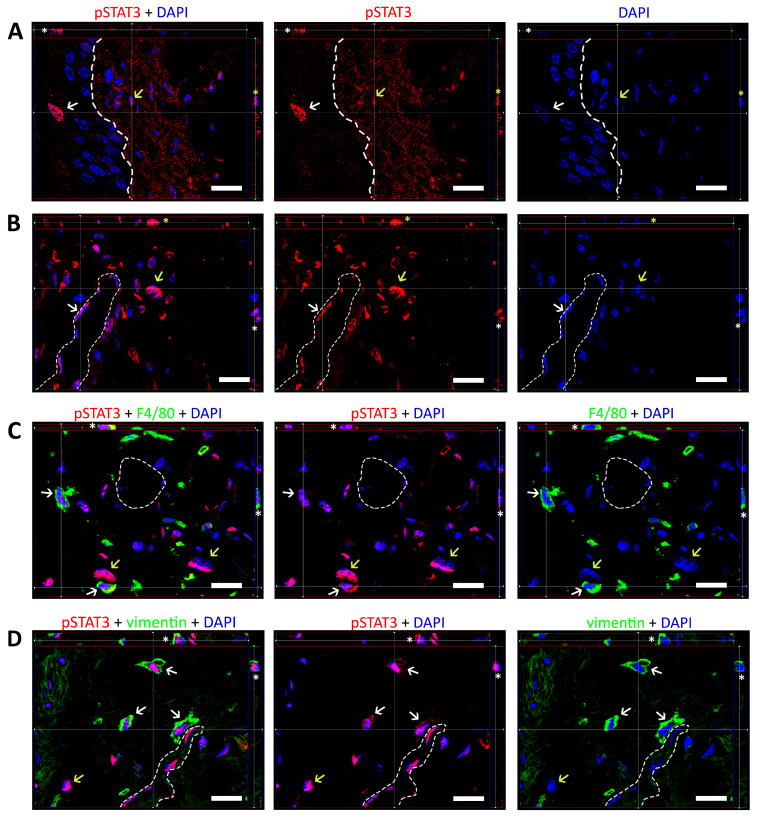
Representative confocal microscopy images of pSTAT3 nuclear translocation in the bladders of CYP-treated mice, selected from a series of images assembling a Z-stack. (**A**) Nuclear expression of pSTAT3 in superficial urothelial cells (white arrows) and interstitial cells of ULP (yellow arrows). A cut-view of the Z-stack shows pSTAT3 immunoreactions in the nucleus of a superficial urothelial cell (white asterisk) and an interstitial cell of the ULP (yellow asterisk). The dotted line indicates the urothelial basal lamina. (**B**) Nuclear expression of pSTAT3 in endothelial cells (white arrows) and the interstitial cells of DLP (yellow arrows). The Z-stack cut-view shows pSTAT3 immunoreactions in the nucleus of the endothelial cell (white asterisk) and interstitial cell of DLP (yellow asterisk). The dotted line indicates the endothelial basal lamina. (**C**) Colocalization of pSTAT3 and F4/80 immunoreactions in some of the interstitial cells of DLP (white arrows). A cut-view of the Z-stack shows pSTAT3 immunoreaction in the nuclei of macrophages (white asterisk). Yellow arrows point to interstitial cells that were not identified as macrophages. The dotted line indicates the endothelial basal lamina. (**D**) Colocalization of pSTAT3 and vimentin immunoreactions in some of the interstitial cells of DLP (white arrows). A cut-view of the Z-stack shows pSTAT3 immunoreactions in vimentin-positive mesenchymal cells (white asterisks). The yellow arrow shows an interstitial cell of non-mesenchymal origin. The dotted line indicates the endothelial basal lamina. Scale bars: 20 µm.

Immunofluorescence labeling revealed nuclear expression of pSTAT3 in individual urothelial cells (Figure 5A), vascular endothelial cells of larger veins located in deep LP (DLP; Figure 5B), and interstitial cells of upper LP (ULP) and DLP (Figure 5A,B). The majority of pSTAT3-positive interstitial cells were located in DLP in close proximity to the larger veins. pSTAT3 and F4/80 (markers of murine macrophages), double-labeled in bladder sections, showed colocalization of both proteins in some of the interstitial cells, identifying them as macrophages (Figure 5C). Double-labelling with vimentin confirmed the nuclear location of pSTAT3 in some of the spindle-shaped cells of mesenchymal origin in the DLP (Figure 5D), identifying them as fibroblasts. In conclusion, we detected nuclear translocation of pSTAT3 in various bladder cells, confirming the activation of the JAK3/STAT3 signaling pathway and involvement of pSTAT3-mediated response in chronic inflammation after CYP-treatment.

### 2.4. Sex Influences the Response of the Bladder Tissue to CYP Treatment

**Figure 6:**
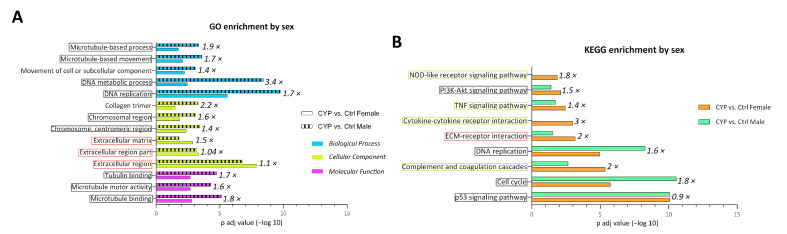
The CYP-induced enrichment of different processes is sex-specific. (**A**) GO enrichment analysis showing significantly enriched biological processes (blue), cell components (yellow), and molecular functions (green). Represented are −log10 *p* adj values for each process, comparing CYP males (dashed bars) and CYP females (empty bars). Numbers at the end of each bar represent the fold change between CYP males and CYP females in the *p* adj value of each enriched process. (**B**) KEGG enrichment analysis showing significantly enriched pathways (−log10 of *p* adj) comparing CYP males (green bars) and CYP females (orange bars). Numbers at the end of each bar represent the fold change between CYP males and CYP females in the *p* adj value of each enriched process. Processes involved in cell proliferation are circled in gray; processes of cell-extracellular space communication are circled in pink; and processes of innate immunity are circled in yellow.

Sexual dimorphism is evident in the occurrence and severity of IC/BPS, since it is considered to be more prevalent and severe in women compared to men [23]. To further identify the sex-based differences between male and female mice, the DEGs identified in CYP vs. Ctrl males and CYP vs. Ctrl females were analyzed separately using enrichment analysis (GO and KEGG). The complete results of GO and KEGG enrichment can be found in Tables S6–S9, showing the enriched pathways with *p* adj < 0.05. Here, we focused only on the processes identified as enriched in both groups, CYP males and CYP females. The fold change between the *p* adj values for each enriched process was calculated for easier representation of differences between sexes (Figure 6A,B). The enrichment analysis comparing Ctrl males vs. Ctrl females did not yield any relevant data (Tables S10 and S11).

The majority of enriched processes that were identified by GO analysis in both CYP males and females were related to cell proliferation or cell division and cell-extracellular space communication (Figure 6A). Similarly, after the exclusion of pathways connected with infectious diseases, non-bladder-related pathologies, and cancer that were identified by KEGG analysis, the remaining pathways enriched in both CYP males and females were related to cell proliferation, innate immunity response, and cell-extracellular space communication (Figure 6B). Two pathways of innate immunity (cytokine-cytokine receptor interaction and NOD-like receptor signaling) were enriched only in the bladders of CYP-treated females and were therefore also included in further analysis. Based on the calculated difference in the fold change of *p* adj values for each pathway between CYP males and females, we can conclude that a sex-specific response to CYP-induced bladder injury exists, which was analyzed in more detail in further experiments.

#### 2.4.1. Processes of Innate Immunity and Tissue Reorganization Are More Enriched in CYP-Treated Females Compared to Males

**Figure 7:**
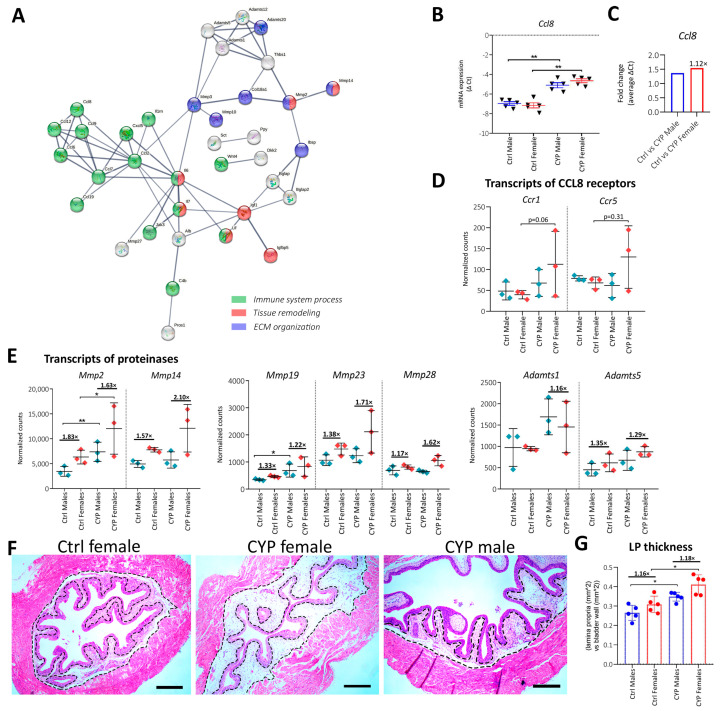
CYP-induced changes in the bladders of female mice. (**A**) STRING network shows interactions between the putative proteins of the significantly DEGs identified in the enriched GO cellular components of the extracellular region, extracellular matrix, and extracellular region part (*n* = 56), displaying only interactions of high confidence (0.7). The proteins are known and predicted to interact in immune system processes (green), tissue remodeling (red), and EMC organization (blue). Disconnected nodes are hidden. (**B**) The mRNA expression of *Ccl8* shows significant upregulation of the transcript in females after CYP treatment. Shown are the means ± SD of negative Δ Ct determined for five animals per group. (**C**) Differences in expression of *Ccl8* mRNA in males and females after CYP treatment. The fold change of average Δ Ct in Ctrl vs. CYP males and Ctrl vs. CYP females is represented on the y axis. The number written above the bars indicates the fold change in mRNA expression in females vs. males after CYP treatment. (**D**) mRNA expression of *Ccr1* and *Ccr5* demonstrates a greater increase in females compared to males after CYP treatment. Shown are the mean ± SD of normalized read counts determined by RNA seq for three animals per group. (**E**) mRNA expression of selected proteinases (*Mmp2*, *Mmp14*, *Mmp19*, *Mmp23*, *Mmp28*, *Adamts1*, *Adamts5*) demonstrates a greater increase in females before and after CYP treatment compared to males. Shown are the mean ± SD of normalized read counts, determined by RNA seq for three animals per group. Blue lines indicate the fold change in average read counts between the groups (Ctrl males vs. Ctrl females and CYP males vs. CYP females). (**F**) Representative images of HE-stained bladder sections showing the difference in LP thickness between Ctrl female, CYP female, and CYP male. Dotted-line borders the measured surface area of LP. Scale bars: 250 µm. (**G**) Quantitative analysis of LP thickness reveals a greater thickness of LP in females compared to males before and after CYP treatment. Blue lines indicate the fold change in average LP thickness between the groups (Ctrl males vs. Ctrl females and CYP males vs. CYP females). * *p* < 0.05; ** *p* < 0.01.

The comparison of *p* adj values of GO-enriched processes between CYP males and females showed a greater enrichment of processes involved in cell-extracellular matrix communication in CYP females compared to CYP males. More specifically, the extracellular region was 1.16-fold and the extracellular matrix was 1.57-fold more enriched in females than in males, while the extracellular region part was similarly enriched in both sexes (Figure 6A). For easier visualization, the DEGs identified in the three pathways (56 altogether) were presented as a STRING network, showing the involvement of their putative proteins in (i) immune system processes, (ii) tissue remodeling, and (iii) extracellular matrix organization (Figure 7A).

The results of GO are consistent with the results of KEGG enrichment, in which the majority of pathways were determined to be more enriched in CYP females compared to CYP males and involved in the processes of innate immunity. Specifically, complement and coagulation cascades and the TNF signaling pathway were 2.02-fold and 1.49-fold more enriched in CYP females, respectively. Interestingly, cytokine-cytokine receptor interaction and the NOD-like receptor signaling pathway were among those significantly enriched in CYP females, while they were significantly downregulated or not deregulated in CYP males (Figure 6B). The comparison of previously obtained qPCR data for genes involved in innate immunity pathways (referred to Section 2.3) by sex confirmed a significant increase in the mRNA expression of *Ccl8* in female bladders after CYP treatment (Figure 7B). The increase was 1.12-fold higher in CYP females compared to CYP males (based on average Δ Ct values; Figure 7C). There were no differences detected in the fold change of the average Δ Ct between sexes for other analyzed genes (Figure S1A). Interestingly, RNA seq data also showed upregulated mRNA expression of *Ccr1* and *Ccr5*, which are the putative receptors of CCL8 [24,25] in the bladders of CYP-treated females, while the receptors were not deregulated in male bladders (Figure 7D). In this regard, CCL8 and its related receptors may represent interesting targets for further research of the sex-dependent immune response in IC/BPS.

In addition to innate immunity pathways, the majority of the DEGs identified as more enriched in CYP females were involved in tissue remodeling and extracellular matrix (ECM) organization (Figure 7A), including different proteases such as matrix metalloproteinases (MMPs) and disintegrin and metalloproteinases (ADAMTs). RNA seq data presented in Figure 7E show higher expression of all proteinase transcripts (*Mmp2*, *Mmp14*, *Mmp19*, *Mmp23*, *Mmp28*, and *Adamts5*) except *Adamts1* in the bladders of females compared to males before and after CYP-treatment (comparing fold change of average normalized counts).

Since MMPs play a major role in the degradation of the ECM components and subsequent loosening of the ECM, which can be evident as expansion of LP [26,27], we analyzed the change in LP thickness after CYP treatment. The increase in LP thickness was significant in both males (*p* = 0.015) and females (*p* = 0.031) after CYP treatment (Figure 7G). However, the measured surface area of LP was 1.18-fold greater in CYP-treated females than in CYP-treated males (Figure 7F,G). The lower density of LP components observed in females may contribute to facilitated infiltration of immune cells, increased availability of cytokines and other pro-inflammatory molecules, and thus to pronounced and more persistent inflammation in the bladder wall.

#### 2.4.2. Cell Proliferation Is More Pronounced in the Urothelium of CYP-Treated Males Compared to Females

**Figure 8:**
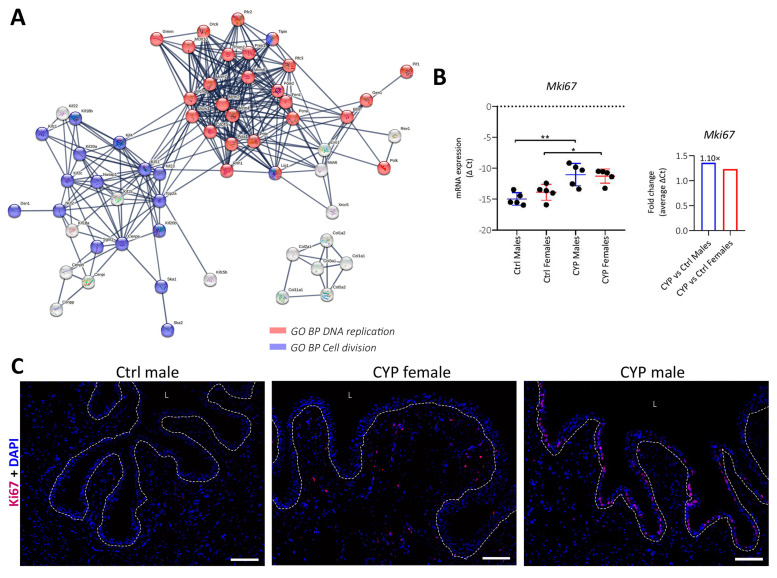
CYP-induced changes in the bladders of male mice. (**A**) STRING network shows interactions between the putative proteins of the significantly DEGs identified in the enriched GO biological processes involved in cell proliferation (*n* = 76), displaying only interactions of the highest confidence (0.9). The proteins are known and predicted to interact during DNA replication (red) and cell division (blue). Disconnected nodes are hidden. (**B**) Left part of the panel: mRNA expression of *Mki67* showing more significant upregulation of the transcripts in males compared to females after CYP treatment. Shown are the means ± SD of negative Δ Ct determined for five animals per group. Right part of the panel: fold change of *Mki67* mRNA expression in males and females after CYP treatment. (**C**) Representative images of Ki67 immunolabeled sections of the bladder wall of Ctrl male, CYP female, and CYP male show the differences in the distribution of Ki67-positive nuclei in the bladder wall. The dotted line represents the basal lamina. L; lumen of the bladder. Scale bar: 100 µm. * *p* < 0.05; ** *p* < 0.01.

The comparison of *p* adj values of GO-enriched processes between CYP males and females showed a greater enrichment of cell division-related processes in males compared to females (Figure 6A). For easier visualization, the DEGs identified in these processes (76 altogether) were presented as a STRING network, showing the involvement of their putative proteins in (i) DNA replication and (ii) cell division (Figure 8A). These results are consistent with the KEGG enrichment analysis, which showed 1.8-fold and 1.6-fold higher enrichment for cell cycle and DNA replication pathways, respectively, in males compared to females (Figure 6B).

Based on these results, we analyzed the expression of a widely used marker of proliferation, Ki67, in our whole bladder tissue samples. qPCR analysis of the Ki67 gene (*Mki67*) showed a significant increase in mRNA expression in both CYP-treated males (*p* = 0.007) and females (*p* = 0.015) compared to Ctrls, with the increase in expression being 1.1-fold higher in CYP-treated males than in CYP-treated females (based on average Δ Ct values; Figure 8B). The analysis of Ki67-immunolabeled sections of the bladder wall revealed a variable distribution of proliferatively active cells in the bladder wall. The majority of Ki67-positive nuclei were detected in the urothelium of CYP-treated males, while in CYP-treated females they were found predominately in the LP (Figure 8D). These data suggest a lower regenerative potential of the female urothelium compared to the male and could be one of the reasons for the higher incidence of IC/BPS in women.

## 3. Discussion

To date, the pathophysiology of IC/BPS remains largely unexplained, as it is a heterogeneous and complex disorder with unspecific clinical symptoms and pathologic findings that often overlap with other bladder-related pathologies [3]. Transcriptome profiling has become invaluable for research in IC/BPS as it is helping to elucidate the molecular mechanisms involved in disease development and progression. Animal models of IC/BPS still remain the main tool in the preclinical research of IC/BPS with an aim to discover novel diagnostic markers and therapeutic targets. Therefore, a detailed understanding of the models is crucial for successful interpretation and translation of the obtained data to the clinical setting. One of the most cited models of IC/BPS is the CYP-induced mouse model [28]. In our study, we created a mouse model mimicking IC/BPS through repeated low-dose CYP applications. The model was analyzed from the transcriptome to protein expression using RNA seq, qPCR, Western blot, and immunohistochemistry. We specifically focused on processes related to inflammatory and immune responses and showed the presence of sex-specific differences that importantly contribute to bladder tissue response to CYP-induced injury. To our knowledge, this is the first study implementing RNA seq technology in the analysis of an IC/BPS murine model induced with multiple CYP applications, which included animals of both sexes.

A complete transcriptome analysis of the bladder tissue collected from CYP-treated C57BL/6J animals showed that CYP treatment significantly alters the transcriptome profile of urinary bladders. Enrichment analysis showed a significant increase in pathways involved in inflammation and cell cycle regulation in the CYP-treated animals. Increments in immune and inflammatory processes are an important finding in the CYP-induced cystitis in rodents [7,29,30], as well as in the bladder samples of patients with IC/BPS [2]. Enrichment in cell cycle regulation was consistent with the increased proliferative status of rodent bladder tissue as a consequence of CYP-caused damage [7] and has also been reflected in the transcriptome of human patients with non-Hunner IC/BPS [13]. In this study, among the immune system-related processes, the innate immunity response was significantly enriched in mouse bladders after CYP treatment. The identified innate immunity pathways included cytokine-cytokine receptor interaction, natural killer cell-mediated cytotoxicity, TNF signaling, complement and coagulation cascades, TLR signaling, and JAK/STAT signaling. These results go hand in hand with the results of transcriptome profiling of bladder samples from patients with non-Hunner [13] and Hunner-type IC/BPS [12], as well as with the results of our previous study of an in vitro IC/BPS model [31], in which the same pathways were identified as significantly enriched. These data clearly demonstrate that our CYP-induced model of chronic bladder inflammation convincingly mimics the inflammatory changes in patient bladders with IC/BPS and support its usefulness as a suitable animal model for understanding IC/BPS mechanisms.

In our subsequent validation analysis by qPCR, we confirmed significantly upregulated mRNA expression of the majority of DEGs involved in innate immunity pathways, identified by RNA seq. Some of them have already been analyzed in previous studies performed on patients and experimental models of IC/BPS and identified as potential diagnostic markers or therapeutic targets. For example, VEGF-D has been shown to be increased in the urine of IC/BPS patients [32], and caspase-8-mediated apoptosis of urothelial cells has been detected in a mouse model of neurogenic cystitis [33]. Some other transcripts confirmed to be upregulated in our study (including *Cd80*, *Eda2r*, and *Osmr*) have never been associated with IC/BPS before. However, their involvement in the development of other diseases such as bladder cancer [34,35] and inflammatory bowel disease is well documented [36,37].

The involvement of JAK/STAT signaling in the development of IC/BPS pathology has only recently come into the focus of preclinical research. Up until now, there have been only a few studies on this topic. Most of them were performed on rat models of IC/BPS, managing to identify the proteins of the JAK/STAT pathway as promising therapeutic targets in IC/BPS [29,38,39,40]. Our study is the first to investigate the JAK/STAT signaling pathway in a CYP-induced mouse model of IC/BPS. The RNA seq data showed significant enrichment of the JAK/STAT pathway, whereas the downstream validation of the identified DEGs revealed JAK3 and STAT3 as the key signaling molecules in CYP-induced chronic cystitis. Increased expression of pSTAT3 after CYP treatment has already been shown in a rat model of cystitis [38], and the inhibition of JAK3 activity has been proven beneficial in reducing the CYP-induced inflammation in rat ulcerative cystitis [40]. Undoubtedly, the involvement of the JAK/STAT pathway in bladder pathologies deserves further investigation, as the deactivation of the IL6/STAT3 signaling has been shown to impair the antimicrobial response in urinary tract infection [41].

The JAK/STAT signaling pathway activated by various cytokines has been previously demonstrated in several cell types, including cells of the immune system [42,43,44], fibroblasts [45], and epithelial cells [46]. In the present study, the nuclear location of pSTAT3 was confirmed in urothelial cells, cells of vascular endothelium, and interstitial cells, mainly located in DLP. The urothelial STAT3 activation is consistent with previous findings in a mouse model of bacterial cystitis [41]. Activation of STAT3 in vascular endothelium has been demonstrated as one of the major contributors to the development of different cardiovascular pathologies [47,48]. Interstitial cells, however, require more careful interpretation. A study by Su and colleagues investigating the immune microenvironment in the urinary bladders of patients with IC/BPS identified tissue-resident macrophages and inflammatory fibroblasts located in the bladder mucosa as key mediators of IC/BPS pathogenesis [19]. We detected both macrophages and fibroblasts in the LP of mouse bladders, and based on their pro-inflammatory role in the IC/BPS pathogenesis, the nuclear location of pSTAT3 in these cells was expected. Immunolabeling confirmed the presence of pSTAT3 in the nuclei of both cell types. Fibroblast activation under inflammatory conditions has previously been extensively studied, including the LIF- and OSM-induced activation via STAT3 [49]. Additionally, STAT3 signaling is presumed to be important for monocyte-to-macrophage differentiation and their polarization into pro-inflammatory or resolving types of macrophages, depending on their microenvironment [47,50]. In this regard, a more detailed role of STAT3-induced macrophage or fibroblast activation in IC/BPS pathology is yet to be discovered.

Sex-specific differences in immune response between males and females have already been identified, suggesting that enhanced innate and adaptive immune responses in females can lead to diminished susceptibility to infections but a greater tendency for the development of autoimmune pathologies [51]. These findings have been reproduced in mice, showing different immune cell profiles between the sexes [52,53]. Moreover, the impact of sexual dimorphism on combating urinary tract infections (UTI) has already been established [54], indicating that sex-specific mechanisms are involved in the development of bladder pathology, implying an urgent need for sex-specific therapeutic strategies. However, studies of various bladder pathologies, including IC/BPS, conducted on patients or animals of both sexes are still scarce.

The present study confirmed sex-specific differences in the immune response of the bladder to CYP injury. Our results point to greater enrichment of innate immunity pathways in CYP-treated females compared to CYP-treated males. Using the applied DEG validation methods, we demonstrated upregulated mRNA expression of *Ccl8* in CYP-treated females compared to CYP-treated males. CCL8 (chemokine ligand 8) acts as a monocyte chemoattractant protein (alternative gene name *Mcp2*) and is mainly expressed by monocytes in healthy mouse bladders according to the scRNA analysis performed by Yu and colleagues [55], but its presence and role in IC/BPS have never been studied. In a mouse colitis model, CCL8 has been shown to be excreted exclusively by CD169+ macrophages, which in turn aids in the recruitment of monocytes to the LP under inflammatory conditions [56]. Interestingly, our RNA seq data showed a significant upregulation of CD169 (gene *Siglec1*, Figure S1B) transcripts, especially in CYP females, that could be the source of increased expression of *Ccl8* in female bladders. Furthermore, the interaction between CCL8 and its putative receptor, CCR5, in the spinal cord has been shown to influence the development of visceral hyperalgesia in a murine colitis model [57], which could potentially also be the cause of persistent pain found in IC/BPS. Therefore, CCL8 may represent an interesting target for the diagnosis and treatment of IC/BPS in the future.

In addition to innate immune responses, sex-specific differences were also found in tissue remodeling and ECM organization that were more pronounced in CYP females compared to CYP males. Both processes could be reflected in a greater thickness of LP in CYP females compared to CYP males due to the degradation of ECM components and fluid accumulation leading to edema. This is a common pathohistological feature of bladders from patients with IC/BPS [58,59], observed also in experimental models [7,60]. Both of the aforementioned processes can be a result of the activity of MMPs [26,61]. Indeed, our RNA seq data showed increased expression of several different MMPs in females compared to males, while quantitative analysis of LP thickness revealed significantly more expanded LP in CYP females in contrast to CYP males. It has been previously reported that sex-dependent expression of MMPs correlates with the tendency for the development of different cardiovascular and neurological diseases [62], while there are no reports in the literature on this topic in bladder pathologies. MMP-induced degradation of ECM components and subsequent reduction in LP density may play an important role in the release of signaling molecules such as cytokines and growth factors that are otherwise bound to ECM components [63]. We therefore hypothesize that the expanded LP in females may lead to a greater availability of cytokines and consequently to a more pronounced inflammatory response in IC/BPS.

Urothelial hyperplasia after CYP treatment is a common finding in IC/BPS rodent models [6,7]; however, this study is the first to elucidate the differences in urothelial cell proliferation between sexes. We found that cell proliferation was more pronounced in the bladders of CYP-treated males compared to CYP-treated females. An interesting finding was the difference in the distribution of Ki67-positive nuclei in the bladder wall, with most of the proliferatively active cells in the urothelium of CYP males but not CYP females. Since urothelial regeneration after injury is imperative for the restoration of normal bladder function and the blood-urine barrier [64,65,66], we assume that the greater ability of urothelial cell proliferation in males may have an important role in the sex-based difference in IC/BPS prevalence. This finding is also in accordance with the diminished urothelial thickness found in predominantly female IC/BPS patients [67], which is associated with decreased proliferation of urothelial cells [68]. However, additional research would be needed to identify possible sexual differences in urothelial regeneration in patients with IC/BPS.

To conclude, this study provides a broad spectrum of data on the most commonly used IC/BPS model, i.e., a mouse model created with multiple low-dose CYP applications. We demonstrated that the CYP treatment significantly alters the bladder transcriptome and results mainly in the enrichment of pathways of innate immunity. Next, we proved the activation of the JAK/STAT signaling system in CYP-treated mice through increased JAK3 phosphorylation and translocation of pSTAT3 to the nuclei of urothelial cells and cells of the LP. We found significantly enriched processes of innate immunity, increased expression of MMPs, and subsequently significantly more pronounced edema of LP in the bladders of CYP females compared to CYP males. In contrast, cell proliferation was a prominent feature of the bladders of CYP-treated males, demonstrating a different regenerative potential of male urothelium compared to females. All these factors together may influence the sex-dependent occurrence of IC/BPS and the severity of symptoms in patients with IC/BPS. Overall, this study represents an invaluable reference dataset for further preclinical research on IC/BPS and greatly expands our current knowledge of the mechanisms involved in disease development. Our study also provides an additional insight into the sex-driven mechanisms and pathways involved in the development and progression of IC/BPS, which may explain why the disease affects women more often than men. Our findings may also serve as a good basis for further research focusing on sex-related differences in cystitis, which could identify sex-specific therapeutic strategies that would be more efficient for the treatment of IC/BPS than those currently used.

## 4. Materials and Methods

### 4.1. Animal Model

Adult (12–14-week-old) C57BL/6J mice (Jackson Laboratory, Bar Harbor, ME, USA) of both sexes (10 males and 10 females; *n* = 20) weighing 20–30 g were included in the study. The animals were housed in polyacrylamide cages in groups of five under constant humidity (55%) and temperature (22 °C) in a 12/12 h light cycle with water and food ad libitum. Prior to the experiment, the animals were allowed an acclimatization period of 14 days. For the experiment, mice were randomly divided into two groups: the CYP-treated group (5 males and 5 females; *n* = 10) and the control (Ctrl) group (5 males and 5 females; *n* = 10). In the CYP group, chronic aseptic cystitis was induced as previously described [6,7]. Briefly, the CYP-treated group of mice received 80 mg/kg of CYP (#C0768, Sigma-Aldrich, Merck, Darmstadt, Germany) diluted in sterile saline i.p. 4-times during an 8-day period (on days 0, 2, 4, and 6), while Ctrl animals received corresponding volumes of sterile saline. Both groups of animals were weighed daily and observed for any signs of discomfort or pain. On the eighth day of the experiment, the animals were euthanized by CO₂-asphyxia. Urinary bladders were removed and cut into smaller pieces for further processing and analysis, as described below. All animal experiments were performed in accordance with the Administration of the Republic of Slovenia for Food Safety, the Veterinary Sector, and Plant Protection, permit number U34401-4/2020/10.

### 4.2. Total RNA Extraction

Samples of the whole bladder wall from 20 mice were snap-frozen in liquid nitrogen and processed for RNA extraction. The tissue was first disrupted using TissueLyser LT (Qiagen, Hilden, Germany) and 5 mm stainless steel beads (50 Hz, 2 × 5 min). Total RNA was extracted using QIAzol Lysis Reagent and RNeasy Plus Universal Mini Kit (both from Qiagen, Hilden, Germany) according to the manufacturer’s protocols. Total RNA concentration and purity were measured with the NanoDrop 2000 spectrophotometer (Thermo Fisher Scientific, Waltham, MA, USA).

### 4.3. RNA Seq and Gene Enrichment Analysis

RNA seq for the obtainment of the mRNA transcriptome was performed in samples isolated from 3 CYP males, 3 CYP females, 3 Ctrl males, and 3 Ctrl females (*n* = 12) by Novogene (Sacramento, CA, USA). Prior to library preparation, the integrity of RNA was assessed using the RNA Nano 6000 Assay Kit and the Bioanalyzer 2100 system (Agilent Technologies, Santa Clara, CA, USA). A total amount of 0.5 µg RNA per sample was used as input material for the RNA sample preparations. mRNA was purified from total RNA using poly-T oligo-attached magnetic beads. After random fragmentation, the first-strand cDNA was synthesized using random hexamer primers, followed by the second-strand cDNA synthesis using DNA polymerase I and RNase H. After completing cDNA terminal repair, A-tailing, adapter ligation, size selection, PCR enrichment, and purification, the library quality was assessed using Agilent Bioanalyzer 2100 systems (Agilent Technologies, Santa Clara, CA, USA). Quantified libraries were sequenced to a depth of 26 million reads/sample on an Illumina Novaseq 6000 platform (Illumina, San Diego, CA, USA), generating 150 bp paired-end fastq files. Raw and processed data are available in the NCBI Gene Expression Omnibus database with the accession number GSE221783.

Raw data in fastq format was processed for quality control using Novogene Co., Ltd. in-house perl scripts (Novogene, Sacramento, CA, USA). High-quality reads were mapped to the mouse genome (Ensembl GRCm39) using Hisat2 (v2.0.5). The mapped reads to genes were quantified by FeatureCounts (v1.5.0-p3) and expressed as fragments per kilobase of transcript sequence per million base pairs (FPKM).

Differential expression analysis using the DESeq2 R package (1.20.0) was performed on five groups: CYP animals vs. Ctrl animals (6 samples per group), CYP males vs. Ctrl males (3 samples per group), CYP females vs. Ctrl females (3 samples per group), CYP males vs. CYP females (3 samples per group), and Ctrl males vs. Ctrl females (3 samples per group). The *p* values for differentially expressed genes (DEGs) were obtained using the negative binomial distribution, followed by Benjamini-Hochberg’s procedure for adjustment. The adjusted *p* values < 0.05 were considered as statistically significant.

Enrichment analysis of DEGs was conducted using clusterProfiler (v.3.8.1). Gene ontology (GO) and Kyoto Encyclopedia of Genes and Genomes (KEGG) enrichment analyses were performed. Pathways with adjusted *p* values of < 0.05 were considered significantly enriched. Heatmaps and principal component analysis (PCA) for selected DEGs were designed using the ClustVis web tool [69]. The protein-protein interactions were visualized by searching the STRING protein interaction database [70].

### 4.4. Reverse Transcription and Quantitative Real-Time Polymerase Chain Reaction (qPCR)

qPCR was used as a secondary method for validation of RNA seq-obtained data on the same samples as used for RNA seq and an additional two samples per group (altogether five samples per group of CYP/Ctrl males and CYP/Ctrl females; *n* = 20). From total mRNA, 1 µg, extracted as described above, was transcribed into cDNA using the 1st Strand cDNA Synthesis Kit for RT-PCR (Roche, Basel, Switzerland) according to the manufacturer’s instructions. The qPCR analyses were performed using 5× FIREpol HOT FIREPol EvaGreen qPCR Mix Plus (Solis Biodyne, Tartu, Estonia) and self-designed primers (Integrated DNA Technologies, Coralville, IA, USA) on the LightCycler 480 System (Roche, Basel, Switzerland). Samples were assayed in triplicate in one run of 50 cycles, composed of denaturation (15 s at 95 °C), annealing (20 s at 60–65 °C), and elongation (20 s at 72 °C). Melt curves were generated at the end of the run, resulting in a single peak. Data were analyzed with the comparative Ct method relative to the expression of endogenous control (*L32*) and presented as a negative Δ Ct between the average Ct of the gene of interest and the average Ct of endogenous control. The sequences of primers used are listed in Table S1.

### 4.5. Western Blot

Samples of whole bladder walls from 3 animals per group (3 CYP males, 3 CYP females, 3 Ctrl males, 3 Ctrl females; *n* = 12) were snap-frozen in liquid nitrogen and homogenized using a tissue pulverizer. The homogenate was lysed in ice-cold RIPA buffer (Merck, Kenilworth, NJ, USA), containing a cocktail of protease and phosphatase inhibitors (Thermo Fisher Scientific, Waltham, MA, USA), and centrifuged at 12,000× *g* for 20 min at 4 °C. Total protein concentrations in supernatants were determined using the Pierce BCA Protein Assay Kit (Thermo Fisher Scientific, Waltham, MA, USA). Equal amounts of protein (50 µg/lane) were separated on 4–20% Novex WedgeWell Tris-Glycine Gels (Invitrogen, Carlsbad, CA, USA) and transferred to a nitrocellulose membrane (Sigma-Aldrich, St. Louis, MO, USA). The membranes were blocked in blocking buffer for 2 h at room temperature (RT) and incubated in the suspension of primary antibodies (anti-JAK1, anti-pJAK1, anti-JAK3, anti-pJAK3, anti-STAT3, anti-pSTAT3, and anti-β-actin) overnight (ON) at 4 °C. After washing in 0.1% Tris-buffered saline/Tween 20 (TBS-T), the membranes were incubated in a suspension of secondary antibodies conjugated with horseradish peroxidase for 2 h at RT. The visualization of the protein bands was performed using the SuperSignal West Femto Maximum Sensitivity Substrate on the iBright FL1500 imaging system (both from Thermo Fisher Scientific, Waltham, MA, USA). The iBright Firmware 1.7 software (Thermo Fisher Scientific, Waltham, MA, USA) was used to analyze the densitometry of the protein bands. The obtained values were normalized to β-actin used as a loading control. Details of the buffers and antibodies used in Western blotting are listed in Table S2.

### 4.6. Immunofluorescence Labeling

Samples of the whole bladder wall from 5 animals per group (5 CYP males, 5 CYP females, 5 Ctrl males, 5 Ctrl females; *n* = 20) were fixated in 10% buffered formalin immediately after excision for 2 h at 4 °C, followed by incubation in 30% saccharose ON at 4 °C. The following day, the bladder samples were embedded in tissue freezing medium (Leica Biosystems, Deer Park, IL, USA), frozen, and sectioned into 5 µm sections using a cryostat (Leica Biosystems, Deer Park, IL, USA). The cryosections were then dried for 2 h at RT, washed in phosphate-buffered saline (PBS), permeabilized, and blocked for 2 h at RT. Subsequently, the bladder sections were incubated ON at 4 °C in a suspension of diluted primary antibodies (anti-pSTAT3, Ki67, F4/80, and vimentin). After washing in PBS, the sections were incubated in a suspension of secondary antibodies conjugated with fluorescent probes for 2 h at RT, washed again in PBS, mounted in Vectashield medium with DAPI (Vector Laboratories, Burlingame, CA, USA), and analyzed with an AxioImager.Z1 fluorescent microscope equipped with ApoTome (Carl Zeiss MicroImaging GmbH, München, Germany). Representative images of pSTAT3, F4/80, and vimentin proteins were taken under immersion objective (63×/NA 1.40). Representative images of Ki67 were taken under a 10x objective. Detailed information for the permeabilization, buffers, and antibodies used in immunolabeling is listed in Table S3.

### 4.7. Quantitative Analysis of LP Thickness

Sections of frozen bladder tissue (5 animals per group), obtained as described above, were stained with hematoxylin and eosin (HE) and photographed with a camera (Bresser MikroCam PRO, Rhede, Germany) using the stereo microscope SMZ800 (Nikon Instruments Inc., Melville, NY, USA). The images were analyzed with ImageJ version 1.53e [71]. Specifically, the surface of the bladder wall (urothelium excluded) was measured in mm^2^ using the free-hand tool. The surface of LP was measured separately, followed by the calculation of the ratio between the LP surface area and the surface area of the whole bladder wall ($\frac{LP\;\left(mm^{2}\right)}{whole\;bladder\;wall\;\left(mm^{2}\right)}$). The measurements were performed on four sections per animal, and the average of the obtained ratios was used for further analysis. Representative images of HE-stained sections were taken with the Nikon Eclipse E200 (Nikon Instruments Inc., Minato City, Tokyo, Japan) under a 4× objective.

### 4.8. Statistical Analysis

A statistical analysis of the obtained data was performed using GraphPad Prism version 8.0 (Dotmatics, Boston, MA, USA). The normality of the data distribution was investigated by the Shapiro-Wilk test. Summary statistics are expressed as mean and standard deviation (SD) or medians and 25th–75th percentiles (Q25–Q75). Statistical differences between two groups were calculated using the Mann-Whitney U-test or an unpaired Student’s *t*-test, depending on the normality of the data distribution. All tests were two-tailed, and a *p* value < 0.05 was considered statistically significant.

## Data Availability

The data presented in this study are openly available in the NCBI repository online at https://www.ncbi.nlm.nih.gov/ with accession number GSE221783.

## Supplementary Materials

The following supporting information can be downloaded at: https://www.mdpi.com/article/10.3390/ijms24065758/s1.
