# Supplementary material for: Molecular Profiling of Inflammatory Processes in a Mouse Model of IC/BPS: From the Complete Transcriptome to Major Sex-Related Histological Features of the Urinary Bladder
Source: Int J Mol Sci. 2023 Mar 17;24(6):5758. doi: 10.3390/ijms24065758 (PMC10058956; doi:10.3390/ijms24065758)
Supplement: Supplementary file 1 [file ijms-24-05758-s001.zip › Supplementary table S1_qPCR sequences.pdf]

Supplementary table S1: List of mouse primers used for qPCR

mRNA	Forward sequence (5' -> 3')	Reverse sequence (5' -> 3')
**L32**	CCTCTGGTGAAGCCCAAGATC	TCTGGGTTTCCGCCAGTTT
**Ccl8**	AGGGATTGAGAGGACGCTAG	GGTGACTGGAGCCTTATCTG
**Eda2r**	CTAATGCTATCTGTGGAGACTGTC	GCTCAACTGGAAGGTACACTG
**Il1rl2**	AGACACGAAGGAGAATACAAACC	ACTGTGTACCGAATTTGATCCC
**Lif**	TTCCCATCACCCCTGTAAATG	GAAACGGCTCCCCTTGAG
**Vegfd**	CATCAGTGCCCGAGTTAGTG	ACAGAGTTTCTTGGAATGAGGAC
**Mlkl**	CTTCCTGGAACTTAGGCTATGG	TGCACACGGTTTCCTAGACG
**Icam1**	GTTCTCTAATGTCTCCGAGGC	CTTCAGAGGCAGGAAACAGG
**Casp8**	AACTTCCTAGACTGCAACCG	TCTCAATTCCAACTCGCTCAC
**Pros1**	TCGTGATGGGAAGATTGAAGTTC	GGCCTCCTTAGCTATTTTAATGC
**Cd80**	GAAGACCCTCCTGATAGCAAG	GTTTCTCTGCTTGCCTCATTTC
**Irak4**	GCTGTGTGAACAACACCATCG	TCTCGTGCTGACACGTTGCC
**Il13ra2**	TCCGAGAAGACGATATTTCCTGG	TCTGGCCCTGTGTAACCTTCC
**Il11**	TGGGACATTGGGATCTTTGC	CATTGTACATGCCGGAGGTAG
**Osmr**	CACAGGCTCTTGATGTATGGAG	TCAGTTGGTTTGGCTTCATTTTC
**Il13ra1**	CACCATTCCAGTCTTTGTCGC	ATACTTCTTCCAGTGCAGGG
**Socs2**	GCTCAGTCAAACAGGATGG	CCAGCTGACGTCTTAACGG
**Socs3**	GAAGATTCCGCTGGTACTGAG	GCTGGGTCACTTTCTCATAGG
**Ptpn6**	TCAGATCAACCAGCGACAGG	GTCACAGTCTAGCCCCTTGG
**Jak1**	CAGAGACCCTTCTTCCGAGC	TCCCAAAGTGACCCTCTCCC
**Jak3**	GAGCCAAGTATCCTACCCGC	TGCACCATGATGCTGTCTCC
**Stat3**	ATCTGTGTGACACCAACGACC	ATGAATCTAAAGTGCGGGGG
**Mki67**	AATAACCATCATTGACCGCTCC	TCTTGACCTTCCCCATCAGGG
