# Supplementary material for: Molecular Profiling of Inflammatory Processes in a Mouse Model of IC/BPS: From the Complete Transcriptome to Major Sex-Related Histological Features of the Urinary Bladder
Source: Int J Mol Sci. 2023 Mar 17;24(6):5758. doi: 10.3390/ijms24065758 (PMC10058956; doi:10.3390/ijms24065758)
Supplement: Supplementary file 1 [file ijms-24-05758-s001.zip › Supplementary table S2_WB protocols.pdf]

Supplementary table S2: detailed description of western blot protocols

Primary antibody	Manufacturer	Dilution	Blocking buffer	Primary antibody dilution buffer
anti-JAK1	Cell Signaling Technology, #3344	1:1000	5% nonfat dry milk in TBS-Tween	5% BSA in TBS-Tween
anti-pJAK1	Cell Signaling Technology, #3331	1:500	5% nonfat dry milk in TBS-Tween	5% BSA in TBS-Tween, incubated for 48 h
anti-JAK3	Cell Signaling Technology, #8863	1:1000	5% nonfat dry milk in TBS-Tween	5% BSA in TBS-Tween
anti-pJAK3	Cell Signaling Technology, #5031	1:1000	5% nonfat dry milk in TBS-Tween	5% BSA in TBS-Tween
anti-STAT3	Cell Signaling Technology, #9139	1:1000	5% nonfat dry milk in TBS-Tween	5% nonfat dry milk in TBS-Tween
anti-pSTAT3	Cell Signaling Technology, #9145	1:1000	5% nonfat dry milk in TBS-Tween	5% BSA in TBS-Tween
anti-βactin	Sigma-Aldrich, #A2066	1:2000	5% nonfat dry milk in TBS-Tween	5% BSA in TBS-Tween
**Secondary antibody**	**Manufacturer**	**Dilution**	**Blocking buffer**	**Secondary antibody dilution buffer**
Goat-anti rabbit, HrP conjugated	Sigma-Aldrich, #A6154	1:1000	/	5% BSA in TBS-Tween
Goat-anti mouse, HrP conjugated	Sigma-Aldrich, #A4416	1:1000	/	5% BSA in TBS-Tween
