# Supplementary material for: Molecular Profiling of Inflammatory Processes in a Mouse Model of IC/BPS: From the Complete Transcriptome to Major Sex-Related Histological Features of the Urinary Bladder
Source: Int J Mol Sci. 2023 Mar 17;24(6):5758. doi: 10.3390/ijms24065758 (PMC10058956; doi:10.3390/ijms24065758)
Supplement: Supplementary file 1 [file ijms-24-05758-s001.zip › Supplementary table S3_IF protocols.pdf]

Supplementary table S3: detailed description of immunofluorescence protocols

**Primary antibody**	**Manufacturer**	**Dilution**	**Permeabilization**	**Blocking buffer**	**Primary antibody dilution buffer**
anti-pSTAT3	Cell Signaling Technology, #9145	1:100	Ice cold 100% methanol, 10 min	5% normal goat serum + 0.3% Triton X-100 in PBS	1% BSA + 0.3% Triton X-100 in PBS
F4/80	Abcam, #ab6640	1:100	Ice cold 100% methanol, 10 min at -20°C	5% normal goat serum + 0.3% Triton X-100 in PBS	1% BSA + 0.3% Triton X-100 in PBS
Ki67	Abcam, #ab16667	1:100	1% BSA + 0.4% Triton X-100 in PBS, 10 min at RT	10% fetal cow serum + 1% BSA in PBS	1% BSA in PBS
**Secondary antibody**	**Manufacturer**	**Dilution**	**Permeabilization**	**Blocking buffer**	**Secondary antibody dilution buffer**
Goat-anti rabbit, AF555 conjugated	Invitrogen, #A21428	1:400	/	/	1% BSA in PBS
Donkey-anti rat, AF488 conjugated	Invitrogen, #A21208	1:400	/	/	1% BSA in PBS
