# Supplementary figures and images for: Molecular Profiling of Inflammatory Processes in a Mouse Model of IC/BPS: From the Complete Transcriptome to Major Sex-Related Histological Features of the Urinary Bladder
Source: Int J Mol Sci. 2023 Mar 17;24(6):5758. doi: 10.3390/ijms24065758 (PMC10058956; doi:10.3390/ijms24065758)

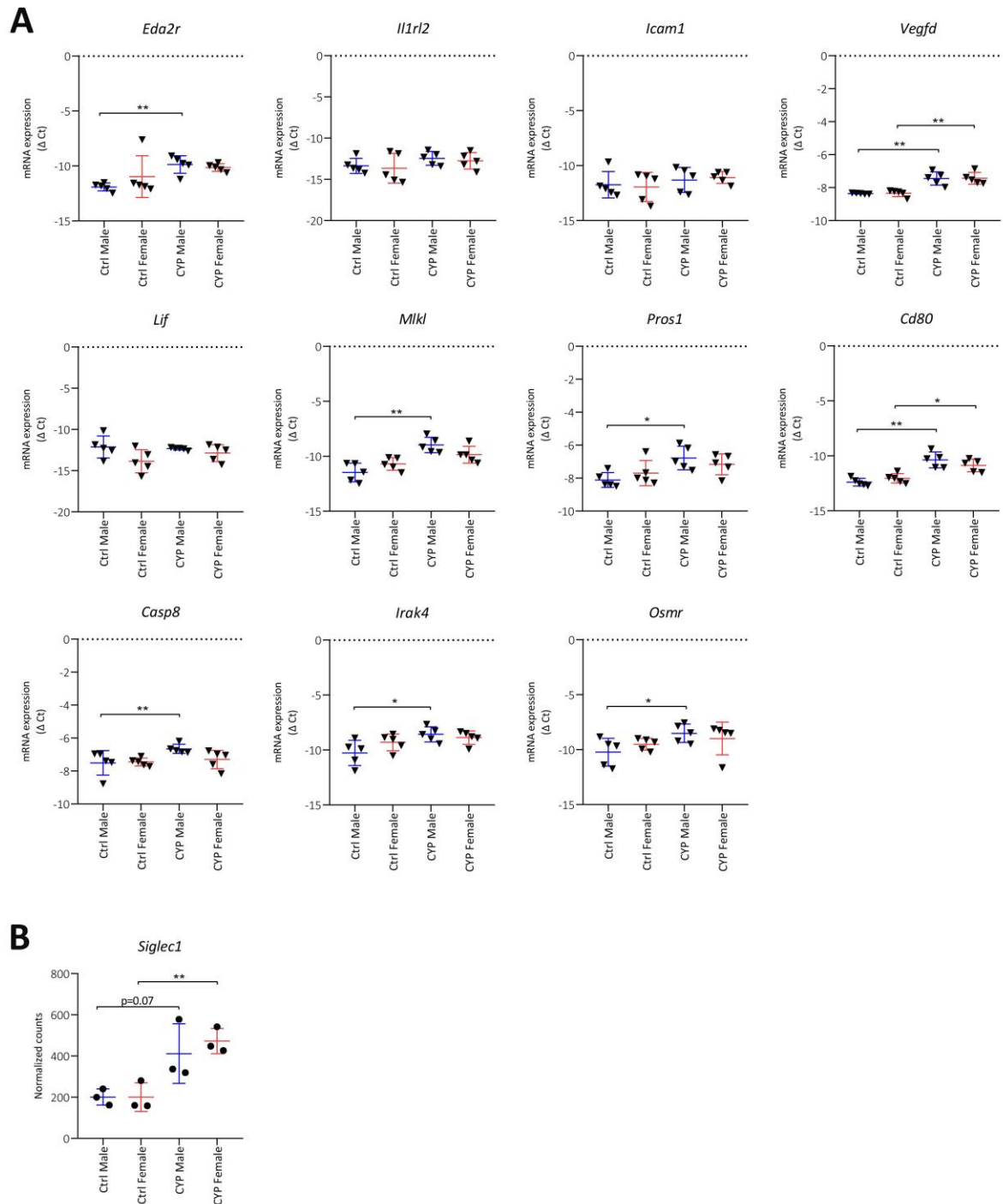

Supplement: Supplementary file 1 [file ijms-24-05758-s001.zip › Supplementary figure S1.pdf]
